# Safety and immunogenicity of BK-SE36/CpG malaria vaccine in healthy Burkinabe adults and children: a phase 1b randomised, controlled, double-blinded, age de-escalation trial

**DOI:** 10.3389/fimmu.2023.1267372

**Published:** 2023-10-16

**Authors:** Alphonse Ouédraogo, Edith Christiane Bougouma, Nirianne Marie Q. Palacpac, Sophie Houard, Issa Nebie, Jean Sawadogo, Gloria D. Berges, Issiaka Soulama, Amidou Diarra, Denise Hien, Amidou Z. Ouedraogo, Amadou T. Konaté, Seni Kouanda, Akira Myoui, Sachiko Ezoe, Ken J. Ishii, Takanobu Sato, Flavia D’Alessio, Odile Leroy, Alfred B. Tiono, Simon Cousens, Toshihiro Horii, Sodiomon B. Sirima

**Affiliations:** ^1^ Groupe de Recherche Action en Santé (GRAS), Ouagadougou, Burkina Faso; ^2^ Department of Malaria Vaccine Development, Research Institute for Microbial Diseases, Osaka University, Suita, Japan; ^3^ European Vaccine Initiative (EVI), Universitäts Klinikum Heidelberg, Heidelberg, Germany; ^4^ Hôpital Protestant Schiphra, Ouagadougou, Burkina Faso; ^5^ Institut de Recherche en Sciences de la Santé, Ouagadougou, Burkina Faso; ^6^ Medical Center for Translational Research, Osaka University Hospital, Suita, Japan; ^7^ Department of Space Infection Control, Graduate School of Medicine, Division of Health Sciences, Osaka University, Osaka, Japan; ^8^ Center for Vaccine and Adjuvant Research, National Institutes of Biomedical Innovation, Health and Nutrition, Ibaraki, Japan; ^9^ Laboratory of Vaccine Science, Immunology Frontier Research Center, Osaka University, Suita, Japan; ^10^ Division of Vaccine Science, Department of Microbiology and Immunology, The Institute of Medical Science, The University of Tokyo, Tokyo, Japan; ^11^ Research and Development Division, Nobelpharma Co., Ltd., Tokyo, Japan; ^12^ Department of Infectious Disease Epidemiology, London School of Hygiene and Tropical Medicine (LSHTM), London, United Kingdom

**Keywords:** BK-SE36/CpG, malaria vaccine, *Plasmodium falciparum*, serine repeat antigen, SERA5, safety, immunogenicity

## Abstract

**Background:**

BK-SE36/CpG is a recombinant blood-stage malaria vaccine candidate based on the N-terminal *Plasmodium falciparum* serine repeat antigen5 (SE36), adsorbed to aluminium hydroxide gel and reconstituted, prior to administration, with synthetic oligodeoxynucleotides bearing CpG motifs. In healthy Japanese adult males, BK-SE36/CpG was well tolerated. This study assessed its safety and immunogenicity in healthy malaria-exposed African adults and children.

**Methods:**

A double-blind, randomised, controlled, age de-escalating clinical trial was conducted in an urban area of Ouagadougou, Burkina Faso. Healthy participants (n=135) aged 21-45 years (Cohort 1), 5-10 years (Cohort 2) and 12-24 months (Cohort 3) were randomised to receive three vaccine doses (Day 0, 28 and 112) of BK-SE36/CpG or rabies vaccine by intramuscular injection.

**Results:**

One hundred thirty-four of 135 (99.2%) subjects received all three scheduled vaccine doses. Vaccinations were well tolerated with no related Grade 3 (severe) adverse events (AEs). Pain/limitation of limb movement, headache in adults and fever in younger children (all mild to moderate in intensity) were the most frequently observed local and systemic AEs. Eighty-three of BK-SE36/CpG (91%) recipients and 37 of control subjects (84%) had Grade 1/2 events within 28 days post vaccination. Events considered by the investigator to be vaccine related were experienced by 38% and 14% of subjects in BK-SE36/CpG and control arms, respectively. Throughout the trial, six Grade 3 events (in 4 subjects), not related to vaccination, were recorded in the BK-SE36/CpG arm: 5 events (in 3 subjects) within 28 days of vaccination. All serious adverse events (SAEs) (n=5) were due to severe malaria (52-226 days post vaccination) and not related to vaccination. In all cohorts, BK-SE36/CpG arm had higher antibody titres after Dose 3 than after Dose 2. Younger cohorts had stronger immune responses (12–24-month-old > 5-10 years-old > 21-45 years-old). Sera predominantly reacted to peptides that lie in intrinsically unstructured regions of SE36. In the control arm, there were no marked fold changes in antibody titres and participants’ sera reacted poorly to all peptides spanning SE36.

**Conclusion:**

BK-SE36/CpG was well-tolerated and immunogenic. These results pave the way for further proof-of-concept studies to demonstrate vaccine efficacy.

**Clinical trial registration:**

https://pactr.samrc.ac.za/TrialDisplay.aspx?TrialID=1921, PACTR201701001921166.

## 1 Introduction

Malaria is one of the oldest human parasitic disease that remains, to date, a major cause of childhood illness and death in sub-Saharan Africa (1, 2). Of the 247 million cases and 619,000 deaths reported in 2021, children younger than 5 years made up 76% of deaths – a percentage that has remained relatively unchanged since 2015 (3, 4). Current malaria interventions and tools, *i.e*., diagnostic and early treatment of cases, malaria chemoprevention, and vector control (3), remain insufficient to achieve the goal of malaria elimination set in the WHO “Global Technical Strategy for Malaria 2016-2030” (5).

Vaccines are one of the most cost-effective and powerful tools for controlling, eliminating, and eradicating infectious diseases. In October 2021, WHO recommended that the RTS,S vaccine be used for the prevention of *P. falciparum* malaria in children from 5 months of age living in regions with moderate to high transmission (4, 6). Although a public health milestone, the vaccine has modest efficacy and provides only short-term protection (7–10). Recently, another anti-sporozoite vaccine, R21/Matrix-M, has been gaining attention based on a higher protective efficacy obtained in a phase 1/2b clinical trial (11). A gap in this arsenal is a vaccine that targets blood-stage parasites to control/limit morbidity.

Unlike the progress in pre-erythrocytic vaccines, advancement in erythrocytic or blood-stage vaccines has been slow and challenging. These vaccines target the highly regulated process of parasite egress and invasion, when a large number of merozoites on a very short time frame are freely available outside the red blood cells. Several blood-stage antigens have reached clinical development, but trials have faltered because of limited efficacy, partly due to antigenic diversity/polymorphism, involvement of functionally redundant pathways, and some safety concerns: erythrocyte-binding antigen-175 (EBA-175), apical membrane antigen-1 (AMA-1), glutamate-rich protein (GLURP), and merozoite surface proteins (MSPs) (12, 13). A phase 1b trial of the viral vectored reticulocyte-binding protein homologue (ChAd63-MVA RH5) has recently been reported (14). Rh5 is a highly conserved antigen with limited polymorphism; and the vaccine-induced anti-Rh5 antibodies exhibited cross-strain growth inhibition activity (GIA). A second-generation vaccine formulation, Rh5.1/Matrix-M, is currently under phase 1 trial in Tanzania (ClinicalTrials.gov NCT04318002). Results of clinical trials on other protein subunit or whole parasite vaccines in development are awaited (13).

The *Plasmodium falciparum* serine repeat antigen 5 (SERA5) is an abundant, essential, blood-stage antigen implicated in parasite egress (15–18). Based on epidemiological and *in vitro* studies, a recombinant form of SERA N-terminal domain (SE36) was selected for clinical development and GMP manufacture (15, 19). In earlier clinical trials, SE36 was adsorbed to aluminium hydroxide gel (AHG) to yield BK-SE36 (19–21). AHG was chosen as the adjuvant due to its well-known safety profile and usage in numerous licensed vaccines (22). However, its utility is limited by its weak activation of cell-mediated immunity (23) and lowered immunogenicity in some clinical trials (20, 24). Some individuals presumed to have a longer history of malaria exposure and repeated infections show lower BK-SE36 responsiveness (20). The state of reduced responsiveness or lowered immunogenicity may be due to immune tolerance. This has been observed for other malaria vaccine candidates (25–27). Vaccines targeting intracellular pathogens likely need both humoral and cell-mediated immune responses to achieve high efficacy (12, 13, 28). New adjuvants have been developed to target specific components of the immune response (23). For example, in the case of RTS,S vaccine, the use of alum with monophosphoryl lipid A, induced high antibody titres but failed to induce protection. The same antigen adjuvanted with oil-in-water emulsion containing monophosphoryl lipid A and QS21 (saponin fraction 21 from *Quillaja saponaria*) resulted in increased antibody production as well as partial protection (29). Hence, the use of a combination of adjuvants could be a viable strategy moving forward to improve the immunogenicity of BK-SE36.

In non-human primate studies, synthetic oligonucleotides (ODN) containing unmethylated cytosine guanine dinucleotide (CpG) motifs on a phosphorothioate backbone selectively promoted cellular and humoral immune responses (23). CpG is recognised by TLR9; and in humans, B cells and plasmacytoid dendritic cells express TLR9, and respond to CpG stimulation (30). Activation of innate immunity by TLR9 leads to upregulation of Th1 proinflammatory cytokines and chemokines, supporting induction of both humoral and cellular arms of adaptive immunity (30, 31). To date, there is only one licensed vaccine which is adjuvanted with CpG (Dynavax HBV vaccine, HEPLISAV). Compared to other HBV vaccines administered in a three-dose regimen, the formulation with CpG 1018-ISS successfully stimulated B and NK cells using a two-dose regimen in adults (>18 years) (23).

Among the TLR9 ligands tested as adjuvant (32), CpG-ODN (K3) with BK-SE36 induced stronger humoral and cellular immune responses than BK-SE36 alone at the ratio of antigen to aluminium to CpG of 10:125:500 µg in both *Macaca fascicularis* and *Saimiri sciureus*. The first-in-human clinical trial of BK-SE36 mixed with CpG-ODN (K3) adjuvant (BK-SE36/CpG) was conducted in healthy malaria-naïve Japanese adult males at the Osaka University Hospital (33). BK-SE36/CpG was well tolerated and the induced antibody titres were 11- to 17-fold higher than with BK-SE36 alone. In addition, seroconversion (or the proportion of vaccinated individuals with >2-fold increase in anti-SE36 IgG titres from baseline) was achieved with fewer vaccine administrations compared to BK-SE36. This robust and broader immune response may help overcome immune tolerance in the malaria exposed population. The promising results from the Japan trial supported the conduct of a phase 1b trial in a malaria endemic area of Burkina Faso. This study presents the first safety and immunogenicity data of BK-SE36/CpG across 3 age cohorts (adults aged 21-45 years, children aged 5-10 years and 12-24 months) in malaria exposed individuals.

## 2 Materials and methods

### 2.1 Trial site and population

The study was carried out by Groupe de Recherche Action en Santé (GRAS) and Institut de Recherche en Sciences de la Santé (IRSS), at the Confessional Hospital of Schiphra located in the urban area of Ouagadougou, the capital of Burkina Faso, from May 2018 to April 2020. From 2011-2015, malaria incidence was reported to remain high (≥ 13.7 cases/10,000 person-weeks) with spatial variability and temporal stability related to *Anopheles gambiae*, *Anopheles arabiensis* and *Anopheles funestus* breeding sites (34–36). The rainy season is between June and October. Modelling outcomes using meteorological and malaria incidence data generated evidence for three transmission periods (low: 16.8–29.9 cases/10,000 person-weeks; high: 51.7–84.8 cases/10,000 person-weeks; and intermediate: 26.7–32.2 cases/10,000 person-weeks) (35). There was no implementation of indoor residual spraying in the area, however, seasonal malaria chemoprevention (SMC) was implemented for children under 5 years (36–38) and bednet usage was high (36).

### 2.2 Study design, objectives and study vaccines

The study was a single-centre, double-blind, randomised, controlled, age de-escalating, phase 1b clinical trial (**Supplementary Material**, Protocol Synopsis). There were three age cohorts: Cohort 1 (21-45 years), Cohort 2 (5-10 years), and Cohort 3 (12-24 months). The primary objective was to assess the safety and reactogenicity of 3 full-doses of malaria vaccine candidate BK-SE36/CpG given at Days 0, 28 and 112, via the intramuscular route. One full dose (1.0 mL) contains 100 µg SE36 antigen, 1 mg aluminium, and 1 mg CpG-ODN (K3). The control, Verorab® rabies vaccine, was administered at 0.5 mL/dose (as per manufacturer’s recommendation). Age de-escalation proceeded only after review of safety data by the principal investigator and Local Safety Monitor (LSM) (to proceed to Cohort 2) and Independent Safety Monitoring Committee (from Cohort 2 to proceed to Cohort 3). Due to the absence of safety data for the BK-SE36/CpG formulation in malaria-endemic areas, this phase 1 trial was limited to participants between 12 months and 45 years. Also, as EPI vaccines are given up to 11 months, children below 12 months receiving EPI vaccines were excluded to avoid any vaccine interference. Similarly, adults older than 45 years-old were not included from this trial because of possible co-/multi- morbidities, disabilities which may be aggravated by CpG-driven immune activation (39). Data on CpG-ODNs to inform dose levels that can induce or worsen autoimmunity are wanting.

### 2.3 Screening, enrolment and randomisation

Community sensitisation sessions were organised by the research team to engage the community in the trial. Participants were randomly drawn from the study area Demographic Surveillance System (DSS) and volunteers were invited to the clinical trial site for a screening visit. During the screening visit, after obtaining informed consent from adults, or the parent(s)/guardian(s), and assent from children aged 7 to 10 years, volunteers were assessed to determine if they were eligible to participate. Individuals were eligible for inclusion in the trial if they provided informed consent, were found to be healthy based on medical history and clinical examination, and indicated their intention to reside/stay within the Ouagadougou health region for the study duration (12 months) (**Supplementary Material**, Protocol Synopsis). Adult volunteers had to agree to practice contraceptive methods. Enrolled participants were assigned a unique identification number and given an identity card.

The randomisation list was prepared by a statistician independent of the trial. Sequentially numbered opaque, sealed envelopes were provided to the unblinded pharmacy team to ensure treatment concealment. For each cohort, subjects were allocated sequentially to treatment in the order that they presented for vaccination.

### 2.4 Blinding

Double blinding in this trial meant that subjects, parent(s)/guardian(s), all investigators, and the study team responsible for the endpoint evaluation were unaware of the vaccine (BK-SE36/CpG or Verorab®) given to the participant. The only staff aware of the vaccine assignment were those responsible for the storage, preparation, and administration of the vaccines and they had no other role in the study. The randomisation list was password protected and was not disclosed to the team before the database lock. Vaccines were stored at 5 ± 3°C under light protected conditions, in a secured area with access limited to the site pharmacy staff. Vaccines were prepared at the clinical trial site pharmacy by the unblinded pharmacy team under the responsibility of an unblinded pharmacist. Contents of syringes were masked to keep the investigator, participants, or participant parents/guardians blinded.

### 2.5 Assessment of study outcomes: safety, reactogenicity and immunogenicity

Clinic visits and assessments were performed according to the trial protocol (**Supplementary Material**, Protocol Synopsis). Following each vaccination, participants were observed for an hour to record any immediate local or systemic adverse events/reactions (AEs). Field workers visited the subjects at home for the next 6 days prior to a clinic visit at the 7^th^ day to record solicited or unsolicited local and systemic Adverse Events Following Immunisation (AEFI). Unsolicited AEs were monitored within one month after each vaccination. Fever was defined as tympanic temperature of ≥ 38.0°C (Cohort 2 and 3) or axillary temperature of ≥ 37.5°C (Cohort 1). Serious Adverse Events (SAEs) were monitored throughout the study. Laboratory safety assessments included hematology (RBC, hemoglobin, MCV, MCH, MCHC, platelets, ESR, and WBC with differential counts) and blood biochemistry (AST, ALT, total bilirubin, and creatinine) tests. AE causality/relatedness was assessed by a study clinician and rated according to the protocol as definitely, probably, possibly, unlikely related, or not related. AE grading of laboratory values was done according to the protocol, adapted to the local reference values at the site. Blood samples were also obtained for autoimmune marker analyses (antinuclear antibodies (ANA), anti-double stranded DNA (anti-dsDNA), antineutrophil cytoplasmic antibody (p- and c-ANCA)) as well as immunogenicity assays. Autoimmune marker analyses were outsourced to laboratoire CERBA, France and total IgG titre by ELISA was measured by CMIC Pharma Science Co., Ltd. (Japan) using standardised methodology (19, 20). Mapping of protective epitope(s) by ELISA against overlapping peptides derived from the SE36 protein was conducted at RIMD, Osaka University (Japan) as described (40). Blood smears were scheduled (and/or done as necessary) to obtain information on malaria infection. Four weeks post Dose 2, incidence of clinical malaria in treatment arms was compared using a case definition of *P. falciparum* asexual parasite density ≥ 5000/µL and documented fever.

### 2.6 Sample size

One hundred and thirty-five (135) healthy participants were planned for 3 cohorts of malaria-exposed African adults and children as described above. The sample size was calculated to address the primary objective (safety). In each cohort (assuming n = 29 evaluable participants in the BK-SE36/CpG arm), the probability of seeing at least one SAE was 95% if the underlying risk of an SAE was 10%. If the risk of an SAE was 5% then the probability of seeing at least one SAE was 77%. With 87 vaccinees the probability of detecting at least one SAE was >99% if the risk was 10%; 99% if the risk was 5% and 58% if the risk was 1%.

### 2.7 Statistical analysis

Statistical analyses were performed at the London School of Hygiene and Tropical Medicine in accordance with the statistical analysis plan. The safety analysis presented here included all subjects who received at least one vaccination. Drop-out data was used until the date of discontinuation. Immunogenicity analyses included all subjects who received at least one vaccination. Analyses were performed using Stata version 15 (www.stata.com). Most analyses were descriptive as the sample size was not adequately powered to enable comparisons between arms. Adverse events were reported on a per arm, dose, and cohort basis.

### 2.8 Ethics statement

This study was conducted according to the principles of the current revision of the Declaration of Helsinki 2013 and in full conformity with relevant country regulations and with the ICH guidelines for GCP ((CPMP/ICH/135/95) July 1996 (and its Revision 2, dated 9 November 2016)). Prior to enrolment, the protocol and the informed consent forms were approved by the National Ethical Committee (Comité d’Éthique pour la Recherche en Santé: N°2017/000098/MS/MERSI/CERS) and the National Regulatory Board (Ministry of Health: N°5004520186EC0000) in Burkina Faso. Ethical reviews were also conducted by the London School of Hygiene and Tropical Medicine Research Ethics Committee (United Kingdom; Ref: 12348 – 2) and the Research Institute for Microbial Diseases Ethics Committee (Japan; Ref: 28-12-3). Importation permit (N°201702432/MS/SG/DGPML/DRLP/SHPS/SBA) for the Investigational Products (BK-SE36 and CpG) was obtained from Agence Nationale de Régulation Pharmaceutique (ARPN), Burkina Faso.

## 3 Results

### 3.1 Participant flow

**Figure 1:**
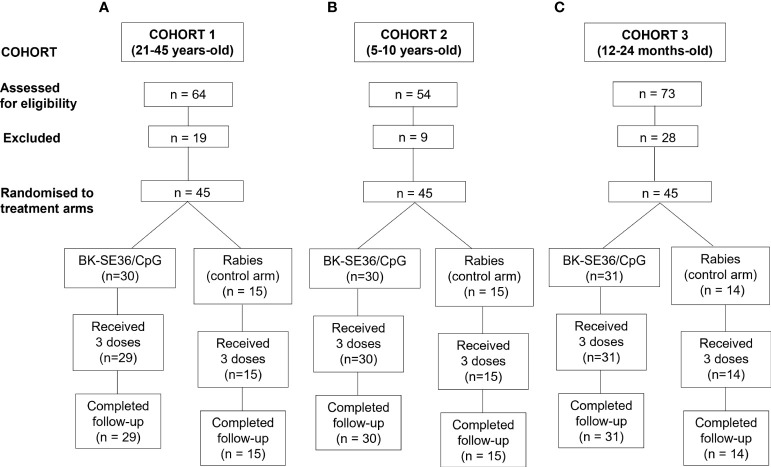
Trial profile for 3 age cohorts. The number of subjects screened, excluded, randomised and included in the final analyses is shown. The age de-escalation GO/No GO criteria were based on safety data. For each participant there was a 4-month vaccination period and a one-year follow-up after the first vaccination. **(A)** Cohort 1, 21-45 years. **(B)** Cohort 2, 5-10 years. **(C)** Cohort 3, 12-24-month-old.

A total of one hundred thirty-five (135) participants were recruited across the three cohorts. All subjects in the 3 cohorts received all 3 scheduled vaccine doses except one (1) subject in the BK-SE36/CpG arm (Cohort 1) who was lost to follow-up (migrated from the study area) after Dose 2. In brief, for Cohort 1 (21-45 years-old), a total of 64 subjects were screened; 19 of whom were assessed as ineligible, while 45 subjects were randomised 2:1 into two study arms with 30 subjects in BK-SE36/CpG and 15 subjects in the control arms (**Figure 1**). The main reasons for ineligibility, alone or in combination, were: not willing to practice contraception (n = 4); ALT >45 U/L (n = 4); blood pressure outside of normal range (n = 4); from outside Ouagadougou (n = 2); low Hb (n = 1); malaria episode (n = 1); peripheral neuropathy (n = 1); neurological pathology (n = 1); and screening to vaccination date exceeded 28 days (n = 1). The safety data of Cohort 1 collected within 7 days after the third vaccination (Dose 3) signaled the start for Cohort 2. For Cohort 2 (5-10 years-old), a total of 54 subjects were screened. Nine volunteers were assessed as ineligible, while 45 subjects were randomised to the two study arms. Reasons for exclusion in this cohort were: consent withdrawn (n = 1); lost to follow-up (n = 1); ALT >45 U/L (n = 1); and sample size already completed (n = 6). After the review of the cumulative safety data from Cohorts 1 and 2, from Day 0 up to 7 days after the completion of the second vaccination of Cohort 2, screening of Cohort 3 started. For Cohort 3 (12-24-month-old), 73 subjects were screened; 28 volunteers were assessed as ineligible, while 31 subjects were assigned to BK-SE36/CpG arm and 14 were assigned to the control arm due to an error in the randomisation list. Reasons for exclusion were: opinion of the investigator (n = 9); Hb <8g/dL (n = 7); severe malnutrition (n = 4); consent withdrawn (n = 2); sample size achieved (n = 2); low platelet count (n = 1); screening to vaccination date exceeded 28 days (n = 1); ALT >45 U/L (n = 1); and participation in another trial (n = 1). The last visit (Day 365) for this cohort was performed 10-20 days earlier in 41/44 subjects to minimize the risk of exposure of participants to the SARS-CoV-2 virus during the COVID-19 pandemic (and in anticipation of unpredictable pandemic timelines). One subject missed the last visit because of parent’s travel and the resulting closed borders.

Study participants in each cohort were comparable at baseline with respect to demography, height, weight, vital signs, and laboratory assessment (**Table 1**).

**Table 1:** Demographic, clinical, and immunological characteristics of enrolled subjects.

	Cohort 1 (21 to 45 years)		Cohort 2 (5 to 10 years)		Cohort 3 (12 to 24 months)	
	BK-SE36/CpG	Rabies	BK-SE36/CpG	Rabies	BK-SE36/CpG	Rabies
Age (years) (SD)	36.5 (5.3)	38.3 (6.9)	7.4 (1.1)	8.1 (1.3)	1.6 (0.2)	1.6 (0.2)
Male (%)	50	60	50	53	61	43
Height (cm) (SD)	167.3 (9.1)	167.3 (8.4)	121.1 (7.4)	124.9 (9.5)	79.7 (4.7)	79.6 (4.4)
Weight (Kg) (SD)	67.2 (12.1)	68.6 (16.7)	20.8 (3.1)	21.6 (3.5)	9.7 (1.1)	9.6 (1.4)
BMI (kg/m^2^) (SD)	24.0 (4.0)	24.7 (6.8)	14.2 (1.2)	13.8 (1.1)	15.3 (1.0)	15.0 (1.0)
Number (%) of participants with detectable IgG titres	23 (77%)	12 (80%)	15 (50%)	9 (60%)	8 (26%)	4 (29%)

SD, standard deviation; BMI, Body Mass Index.

### 3.2 Safety and reactogenicity

From 0 to 28 days post vaccination, AEs were reported in all cohorts, in both treatment arms. Eighty-three subjects that received BK-SE36/CpG (n = 83/91, 91%) had 334 AEs, of which 119 AEs from 35 subjects (38%) were related to vaccination (**Supplementary Table 1**). Thirty-seven subjects that received the control vaccine (n = 37/44, 84%) had 127 AEs, of which 22 AEs from 6 subjects (14%) were related to vaccination. No vaccine-related events were reported as Grade 3 or as SAE in either arm (**Supplementary Table 2**). AEs were more common in the vaccine arm, but almost all of them were Grade 1 events. Six Grade 3 (severe) events, all in the BK-SE36/CpG arm, were reported throughout the trial and were not related to vaccination: 5 events were recorded 7-28 days post vaccination from three subjects (3%); and another at 84 days post vaccination (**Supplementary Table 3**). It is worth noting that 4 of the events were paired events in two individuals: SEK-A08 (Cohort 1, see Section on *AEFIs*) and SEK-T33 (Cohort 3, see Section on *Other AEs*) who both experienced elevated liver enzymes on one occasion each.

#### 3.2.1 Immediate reactogenicity

The most frequent events immediately post vaccination were pain/limitation of limb movement, abnormal blood pressure, abnormal pulse, and headache (**Table 2**). Frequency of signs/symptoms did not appear to vary by treatment arm, number of the dose or the cohorts. Six cases of drowsiness post vaccination were recorded in Cohort 3, post Dose 1 (3 events in BK-SE36/CpG, 1 event in control arm) and Dose 2 (1 event each for BK-SE36/CpG and control arm). All reactions were mild (Grade 1), except for a moderate (Grade 2) headache in the control arm after Dose 3 in Cohort 1.

**Table 2:** Immediate reactogenicity (within the first 60 minutes) after each vaccine dose.

	Cohort 1					
	Dose 1		Dose 2		Dose 3	
	BK-SE36/CpG	Control	BK-SE36/CpG	Control	BK-SE36/CpG	Control
	n = 30	n = 15	n = 30	n = 14	n = 29	n = 15
Abnormal temperature	0	0	0	0	0	0
Abnormal blood pressure	3 (10%) (95% CI 2 to 27%)	0	0	1 (7%) (95% CI 0 to 34%)	0	0
Abnormal pulse	2 (7%) (95% CI 1 to 22%)	2 (13%) (95% CI 2 to 40%)	6 (20%) (95% CI 8 to 39%)	2 (14%) (95% CI 2 to 43%)	2 (7%) (95% CI 1 to 23%)	2 (13%) (95% CI 2 to 40%)
Local swelling present	0	0	0	0	0	0
Local redness present	0	0	0	0	0	0
Local induration present	0	0	0	0	0	0
Pain/limitation of limb movement	0	0	0	1 (7%) (95% CI 0 to 34%)	2 (7%) (95% CI 1 to 23%)	0
Headache	1 (3%) (95% CI 0 to 17%)	0	0	0	1 (3%) (95% CI 0 to 18%)	1 (7%) (95% CI 0 to 32%)
Malaise	0	0	0	0	0	0
Myalgia	0	0	0	0	0	0
Arthralgia	0	0	0	0	0	0
Nausea	0	0	0	0	0	0
Vomiting	0	0	0	0	0	0
Other AE	0	0	0	0	0	0

	Cohort 2					
	n = 30	n = 15	n = 30	n = 15	n = 30	n = 15
Abnormal temperature	0	0	0	0	0	0
Abnormal blood pressure	2 (7%) (95% CI 1 to 22%)	1 (7%) (95% CI 0 to 32%)	0	1 (7%) (95% CI 0 to 32%)	0	0
Abnormal pulse	5 (17%) (95% CI 6 to 35%)	3 (20%) (95% CI 4 to 48%)	2 (7%) (95% CI 1 to 22%)	2 (13%) (95% CI 2 to 40%)	3 (10%) (95% CI 2 to 27%)	0
Local swelling present	0	0	0	0	0	0
Local redness present	0	0	0	0	0	0
Local induration present	0	0	0	0	0	0
Pain/limitation of limb movement	3 (10%) (95% CI 2 to 27%)	1 (7%) (95% CI 0 to 32%)	0	0	3 (10%) (95% CI 2 to 27%)	0
Age ≥ 7 years	n = 16	n = 13	n = 17	n = 13	n = 22	n = 14
Headache	0	1 (8%) (95% CI 0 to 36%)	0	0	0	0
Malaise	0	0	0	0	0	0
Myalgia	0	0	0	0	0	0
Arthralgia	0	0	0	0	0	0
Nausea	0	0	0	0	0	0
Vomiting	0	0	0	0	0	0
Age < 7 years	n = 14	n = 2	n =13	n =2	n = 8	n = 1
Loss of appetite	0	0	0	0	0	0
Irritability/fussiness	0	0	0	0	0	0
Drowsiness	0	0	0	0	0	0
Other AE	0	0	0	0	0	0

	Cohort 3					
	n = 31	n = 14	n = 31	n = 14	n = 31	n = 14
Abnormal temperature	0	1 (7%) (95% CI 0 to 34%)	0	0	0	0
Abnormal blood pressure	3 (10%) (95% CI 2 to 26%)	1 (7%) (95% CI 0 to 34%)	5 (16%) (95% CI 5 to 34%)	1 (7%) (95% CI 0 to 34%)	4 (13%) (95% CI 4 to 30%)	1 (7%) (95% CI 0 to 34%)
Abnormal pulse	8 (26%) (95% CI 12 to 45%)	2 (14%) (95% CI 2 to 43%)	3 (10%) (95% CI 2 to 26%)	5 (36%) (95% CI 13 to 65%)	5 (16%) (95% CI 5 to 34%)	0
Local swelling present	0	0	0	0	0	0
Local redness present	0	0	0	0	0	0
Local induration present	0	0	0	0	0	0
Pain/limitation of limb movement	0	0	0	1 (7%) (95% CI 0 to 34%)	1 (3%) (95% CI 0 to 17%)	0
Loss of appetite	0	0	0	0	0	0
Irritability/fussiness	0	0	0	0	0	0
Drowsiness	3 (10%) (95% CI 2 to 26%)	1 (7%) (95% CI 0 to 34%)	1 (3%) (95% CI 0 to 17%)	1 (7%) (95% CI 0 to 34%)	0	0
Other AE	0	0	0	0	0	0

#### 3.2.2 Seven-day follow-up after each vaccination

Local AEs were mostly pain/limitation of limb movement. Fifty individuals (37%) reported pain/limitation of movement 81 times across the three vaccine doses (**Table 3**). In Cohort 1, aside from pain/limitation of limb movement (22 events from n=14 subjects in BK-SE36/CpG arm and n=1 from control arm), one individual in the BK-SE36/CpG arm reported local swelling after the first vaccine dose. In Cohort 2, there were 51 events of pain/limitation of limb movement (n = 22 from BK-SE36/CpG, n = 7 from control arm); and in Cohort 3, 8 events (n = 6 all from BK-SE36/CpG arm). In total, 42/91 = 46% individuals in the BK-SE36/CpG arm reported local AEs compared with 18% (8/44) in the control arm. All local events were vaccine-related, of short duration (<1-2 days), and mostly mild (except for a moderate (Grade 2) injection site pain reported after Dose 2 in Cohort 3).

**Table 3:** Solicited, vaccine related mild to moderate adverse events on Days 1-7 after each vaccine dose.

	Cohort 1					
	Dose 1		Dose 2		Dose 3	
	BK-SE36/CpG	Control	BK-SE36/CpG	Control	BK-SE36/CpG	Control
	n = 30	n = 15	n = 30	n = 15	n = 29	n = 15
Local						
Local swelling present	1 (3%) (95% CI 0 to 17%)	0	0	0	0	0
Local redness present	0	0	0	0	0	0
Local induration present	0	0	0	0	0	0
Pain/limitation of limb movement	6 (20%) (95% CI 8 to 39%)	0	10 (33%) (95% CI 17 to 53%)	0	5 (17%) (95% CI 6 to 36%)	1 (7%) (95% CI 0 to 32%)
Systemic						
Fever	0	0	0	0	1 (3%) (95% CI 0 to 18%)	0
Headache	1 (3%) (95% CI 0 to 17%)		2 (7%) (95% CI 1 to 22%)	1 (7%) (95% CI 0 to 32%)	2 (7%) (95% CI 1 to 23%)	3 (20%) (95% CI 4 to 48%)
Malaise	0	0	0	0	0	0
Myalgia	0	0	0	0	0	0
Arthralgia	0	0	0	0	0	0
Nausea	0	0	0	0	1 (3%) (95% CI 0 to 18%)	0
Vomiting	0	0	0	0	1 (3%) (95% CI 0 to 18%)	0

	Cohort 2					
	n = 30	n = 15	n = 30	n = 15	n = 30	n = 15
Local						
Local swelling present	0	0	0	0	0	0
Local redness present	0	0	0	0	0	0
Local induration present	0	0	0	0	0	0
Pain/limitation of limb movement	12 (40%) (95% CI 23 to 59%)	3 (20%) (95% CI 4 to 48%)	17 (57%) (95% CI 37 to 75%)	4 (27%) (95% CI 8 to 55%)	15 (50%) (95% CI 31 to 69%)	0
Systemic						
Fever	0	0	0	0	4 (13%) (95% CI 4 to 31%)	0
Age ≥ 7 years	n = 16	n = 13	n = 17	n = 14	n = 22	n = 14
Headache	0	1 (8%) (95% CI 0 to 36%)	0	0	3 (14%) (95% CI 3 to 35%)	0
Malaise	0	0	0	0	0	0
Myalgia	0	0	0	0	0	0
Arthralgia	0	0	0	0	0	0
Nausea	0	0	0	0	0	0
Vomiting	0	0	0	0	0	0
Age < 7 years	n = 14	n = 2	n = 13	n = 1	n = 8	n = 1
Loss of appetite	0	0	0	0	0	0
Irritability/fussiness	0	0	0	0	0	0
Drowsiness	0	0	0	0	0	0

	Cohort 3					
	n = 31	n = 14	n = 31	n = 14	n = 31	n = 14
Local						
Local swelling present	0	0	0	0	0	0
Local redness present	0	0	0	0	0	0
Local induration present	0	0	0	0	0	0
Pain/limitation of limb movement	6 (19%) (95% CI 7 to 37%)	0	2 (6%) (95% CI 1 to 21%)	0	0	0
Systemic						
Fever	9 (29%) (95% CI 14 to 48%)	0	6 (19%) (95% CI 7 to 37%)	1 (7%) (95% CI 0 to 34%)	4 (13%) (95% CI 4 to 30%)	0
Loss of appetite	0	0	0	0	0	0
Irritability/fussiness	0	0	0	0	0	0
Drowsiness	0	0	0	0	0	0

For systemic events, in Cohorts 1 and 2, most events (20 events in total) were headache (13; n = 7 from BK-SE36/CpG arm, n = 4 from control arm). In Cohort 3, fever was reported on 19 occasions in the BK-SE36/CpG arm (n = 13) but only once in the control arm. All were, however, of short duration (≤1 day); and most events were mild except for 3 Grade 2 headache events post Dose 3 in Cohort 1 (n = 1 from BK-SE36/CpG arm, n = 2 from control arm).

#### 3.2.3 Adverse events following immunisation

A total of 290 events were classified as AEFI. Seventy-six individuals (76/135, 56%) in the BK-SE36/CpG arm had 222 events; while 26 subjects (26/135, 19%) in the control group had 68 AEFIs. More subjects in the BK-SE36/CpG arm experienced AEFIs related to vaccination than in the control arm (Cohort 1, BK-SE36/CpG: 30%, 43%, and 27% *vs* control: 7%, 13% and 27%, for Dose 1, 2 and 3, respectively; Cohort 2, BK-SE36/CpG: 40%, 57%, and 53% *vs* control: 27%, 27%, and 0%, for Dose 1, 2 and 3, respectively; Cohort 3, BK-SE36/CpG: 39%, 23%, and 19% *vs* control: 14%, 14%, and 7%, for Dose 1, 2 and 3, respectively). Other than injection site pain, fever and headache, commonly reported events were rhinitis (44), cough (17), diarrhoea (16) and abdominal pain (11). Most of the AEFIs were mild or moderate, except for 2 Grade 3 events (increased alanine aminotransferase; increased aspartate aminotransferase) in a Cohort 1 subject (SEK-A08) after Dose 3. The paired event was unlikely to be related to the vaccine and not clinically significant (**Supplementary Table 3**).

#### 3.2.4 Unsolicited adverse events

Unsolicited AEs were ascertained at each clinic visit from Day 0 to Day 28 post vaccination. Within each cohort, the frequency of events was similar in both treatment arms. Events related to vaccination (n = 19) were mild and most resolved within <1-2 days (**Supplementary Table 4**). An abnormal laboratory value (high serum creatinine) and a mild backpain in Cohort 1, resolved within 21 and 10 days, respectively. Commonly reported events were pyrexia (n = 5), injection site pain (n = 3) and diarrhoea (n = 3). All AEs resolved without sequelae.

#### 3.2.5 Other AEs

Other AEs recorded at any time during the trial across the three cohorts were common illnesses such as rhinitis, cough, pyrexia/fever, diarrhoea, malaria, and headache. All were unlikely or not related to vaccination; most were mild or moderate, although 4 Grade 3 events in the BK-SE36/CpG arm were reported: haemoglobin decrease (n = 2, 28 or 84 days post Dose 2), increases in aspartate (n = 1, 28 days post Dose 3) and alanine (n = 1, 28 days post Dose 3) aminotransferases (**Supplementary Table 3**).

#### 3.2.6 SAEs

No Suspected Unexpected Serious Adverse Reaction (SUSARs) or deaths were reported. There were five SAEs throughout the trial, all due to severe malaria, not related to the vaccine, not life-threatening and did not lead to withdrawal. One (1) SAE was reported in Cohort 1 (21-45 years-old) and four (4) SAEs were reported in Cohort 2 (5-10 years-old). All resolved in less than two weeks.

#### 3.2.7 Laboratory safety tests

Fluctuations in laboratory values, mostly considered not clinically significant, were noted across treatment arms and at various timepoints during the trial.

Clinically significant abnormal values in Cohort 1 were: decreased in neutrophil count (3 events: BK-SE36/CpG, in two subjects (n = 2)), high serum creatinine (2 events: BK-SE36/CpG, n = 1; control, n = 1), high ALT (10 events: 9 from BK-SE36/CpG, n = 5; control, n = 1), high AST (9 events: 8 from BK-SE36/CpG, n = 4; control, n = 1), and high bilirubin (20 events: 9 from BK-SE36/CpG, n = 4; 11 from control, n = 3). Of 44 clinically significant fluctuations, one event (high serum creatinine in a BK-SE36/CpG subject) was possibly related to vaccination (Grade 1, resolved in 21 days) and reported as an unsolicited AE (as above). For Cohort 2 subjects, a decrease in haemoglobin (1 event: BK-SE36/CpG), high ALT (1 event: BK-SE36/CpG) and high AST (3 events: BK-SE36/CpG, n = 2; control, n = 1) were reported. For Cohort 3, decreased haemoglobin (1 event: BK-SE36/CpG), high ALT (4 events: BK-SE36/CpG, n = 3; control, n = 1) and high AST (5 events: 4 from BK-SE36/CpG, n = 3; control, n = 1) were reported. In Cohorts 2 and 3, none of the 5 and 10 clinically significant fluctuations, respectively, were related to vaccination.

**Figure 2:**
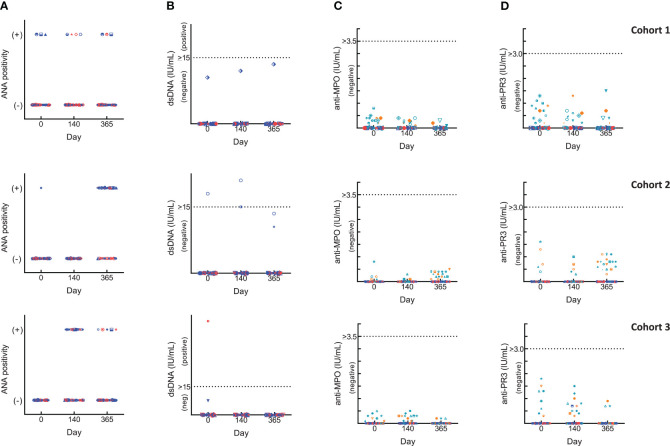
Changes in laboratory autoimmune markers. Blue: BK-SE36 arm; pink: control arm. Day 0: prior to first Dose; Day 140: 4 weeks post Dose 3; Day 365: 1 year post Dose 1. Each row corresponds to a cohort indicated on the left. Results were interpreted according to CERBA Criteria: **(A)** Assessment for ANA: results were categorised based on immunofluorescence testing of the subject’s serum at various dilutions and were reported either as negative (-) or on fluorescent patterns (+: either, speckled, nuclear, homogenous, centromere or NuMa1) observed. **(B)** Assessment for dsDNA: <10 IU/mL, negative; 10-15 IU/mL, assay limit/threshold; >15 IU/mL, positive. **(C, D)** ANCA results were first tested by indirect immunofluorescence. Any titre <20 is treated as negative. When titres are ≥20, the specificity of ANCA was investigated by ELISA to help identify the targeted protein in the neutrophils: cANCA (or cytoplasmic ANCA) targets a protein called proteinase 3 (anti-PR3) and pANCA (or perinuclear ANCA) targets a protein called myeloperoxidase (anti-MPO). Teal: BK-SE36 arm; orange: control arm. **(C)** Assessment for anti-MPO: <3.5 IU/mL, negative; 3.5-5.0 IU/mL, assay limit/threshold; >5.0 IU/mL, positive. **(D)** Assessment for anti-PR3: < 2.0 IU/mL, negative; 2.0-3.0 IU/mL, assay limit/threshold; >3.0 IU/mL, positive.

Changes in autoimmune markers (ANA, anti-dsDNA, p- and c-ANCA) were observed as transient fluctuations, high baseline/early elevations even for those who did not receive BK-SE36/CpG (**Figure 2**). For ANA, some subjects had a positive fluorescence pattern: at baseline (Cohort 1, 10%; Cohort 2, 7%); 4 weeks after Dose 3 (Cohort 1: BK-SE36/CpG, 7%; control, 13%; Cohort 3: BK-SE36/CpG, 39%; control, 29%); and at Day 365 (Cohort 1: BK-SE36/CpG, 7%; control, 7%; Cohort 2: BK-SE36/CpG, 70%; control, 33%; Cohort 3: BK-SE36/CpG, 10%; control, 15%). For dsDNA, in Cohort 1, no subject was positive although one adult in the BK-SE36/CpG arm (3%) had anti-dsDNA levels at assay threshold at all visits, including baseline. A child in Cohort 2, BK-SE36/CpG arm (3%), had a positive anti-dsDNA at Day 0 and 4-weeks post Dose 3. In Cohort 3, a child randomised to the control arm (7%) had high anti-dsDNA at baseline. At Day 365, all subjects were negative for anti-dsDNA.

In all cohorts of both treatment arms, and at any visit some ANCA positive immunofluorescence were detected and as per SOP the specificity of the results were investigated by ANCA screen ELISA test intended for the detection of proteins in neutrophils: anti-myeloperoxidase (anti-MPO, perinuclear ANCA) and/or anti-proteinase 3 (anti-PR3, cytoplasmic ANCA). All testing for both p-ANCA and c-ANCA were negative. Furthermore, no subject was positive for at least two markers at the same scheduled visit and no positive events were associated with a particular clinical diagnosis.

### 3.3 Immunogenicity

#### 3.3.1 Total anti-SE36 IgG response

**Figure 3:**
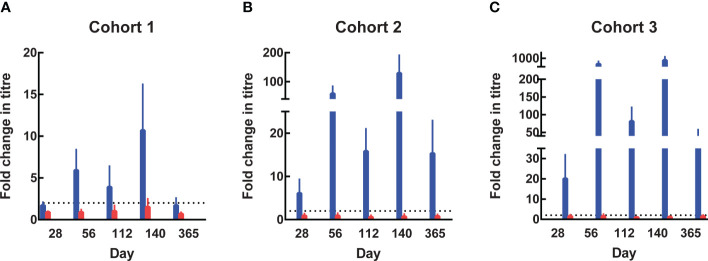
Fold change in antibody titres from Day 0. In the 3 age cohorts, fold change in antibody titres from baseline were calculated by dividing the titre at subsequent visits by the titre at Day 0 (prior to Dose 1). The value of 8 was assigned to undetectable titres when calculating the fold change. **(A)** Cohort 1, 21-45 years. **(B)** Cohort 2, 5-10 years. **(C)** Cohort 3, 12-24-month-old. Blue: BK-SE36 arm; pink: control arm.

Anti-SE36 antibodies were detected before vaccination in both arms across the three cohorts. Four weeks after each vaccination, the BK-SE36/CpG arms had markedly higher mean anti-SE36 antibody titres compared to the control arm (**Table 4**). Mean anti-SE36 antibody titres (GMT) in all cohorts were higher 4 weeks after Dose 3 (Day 140) compared to 4 weeks after Dose 2 (Day 56). A stronger humoral immune response was observed in the younger cohorts: Cohort 3 (12–24-month-old) subjects had higher anti-SE36 IgG titre than Cohort 2 (5-10 years old), who in turn had higher GMT than Cohort 1 (21-45 years old). Only children (5-10 years old and 12-24-month-old) in the BK-SE36/CpG arms had >2-fold change in antibody titres from baseline (Day 0) at any time-point after vaccination (**Figure 3**).

**Table 4:** Total anti-SE36 IgG antibody.

	Trial Arms			
	BK-SE36/CpG		Control (Verorab® rabies vaccine)	
	n	GMT (95% CI)	n	GMT (95% CI)
*Cohort 1*				
**Day 0**	30	79.7 (37.1, 170.9)	15	84.8 (41.9, 171.6)
**Day 28**	30	100.9 (50.5, 201.4)	15	89 (40.9, 193.3)
Day 56	30	272.9 (162, 459.7)	15	78.5 (38.3, 161)
**Day 112**	29	238.3 (119, 477.2)	15	132.3 (55.8, 313.8)
Day 140	29	518.1 (330.2, 813.1)	15	94.4 (44.3, 201.1)
Day 365	29	207 (105.8, 404.8)	15	124.2 (65.9, 234)
*Cohort 2*				
**Day 0**	30	37.3 (27.1, 51.4)	15	44.8 (23.5, 85.4)
**Day 28**	30	105.8 (72.2, 154.9)	15	52.5 (23.6, 116.9)
Day 56	30	1009.3 (721.6, 1411.7)	15	33.4 (19.1, 58.3)
**Day 112**	30	272.5 (218.1, 340.4)	15	98.6 (22, 441.8)
Day 140	30	2231.3 (1704, 2921.8)	15	79.5 (20.5, 307.9)
Day 365	30	263.6 (184.9, 375.8)	15	41.3 (16.6, 103)
*Cohort 3*				
**Day 0**	31	23.5 (14.7, 37.5)	14	21.1 (8.2, 53.9)
**Day 28**	31	265.9 (189, 374.1)	14	36.7 (20.3, 66.5)
Day 56	31	5184.1 (3546.5, 7578)	14	30.5 (18, 51.7)
**Day 112**	31	858.5 (604.1, 1220.2)	14	24 (17.1, 33.9)
Day 140	31	8715.4 (6766.9, 11225.1)	14	23.9 (19, 30.1)
Day 365	31	415.8 (304.7, 567.6)	13	33 (23.9, 45.5)

Subjects were vaccinated at Day 0 (Dose 1), 28 (Dose 2) and 112 (Dose 3). Day 28, 56 and 140 = 4 weeks after Dose 1, 2 and 3, respectively. GMT, geometric mean titre (95% confidence interval); n = no. of subjects.

In Cohort 1 (21-45 years old), the proportion of subjects with detectable anti-SE36 IgG titres prior to vaccination was comparable between treatment arms: 77% (BK-SE36/CpG) *vs* 80% (control). Four weeks post Dose 2, the proportion of BK-SE36/CpG vaccinees with detectable IgG titres was 100%, with a 5.9-geometric mean fold change in antibody titres from Day 0 (**Figure 3A**). Dose 3 induced a 10.7-fold change (*vs* 0.9- and 1.5-fold change, post Doses 2 and 3, respectively, in control subjects). Of note, by Day 365, wanning in antibody titres were observed as only 72% had detectable anti-SE36 IgG titres, with 1.7-fold change from baseline (*vs* 53%, 0.7-fold change for control).

In Cohort 2 (5-10 years old), the proportion of subjects with detectable anti-SE36 IgG titres prior to vaccination was also comparable: 50% (BK-SE36/CpG) *vs* 60% (control). In the BK-SE36/CpG arm, the proportion of participants with detectable IgG titres was 100%, 4 weeks post Dose 1 with 6.1-fold change from Day 0. Doses 2 and 3 induced a 58.4- and 129.2-fold change (*vs* 0.9 and 0.7-fold change, respectively, in control subjects) (**Figure 3B**). At Day 365, 100% of vaccinees still had detectable anti-SE36 IgG titres, with 15.3-fold change from baseline (*vs* 47%, 0.8-fold change for control).

Likewise, in Cohort 3 (12–24-month-old), the proportion of subjects with detectable anti-SE36 IgG titres prior to vaccination was comparable between treatment arms: 26% (BK-SE36/CpG) *vs* 29% (control). Detectable IgG titres in those that received BK-SE36/CpG were 100%, 4 weeks post Dose 2 with 491-fold change from baseline. Dose 3 induced an 825-fold change (*vs* 1.5 and 1.1-fold change, post Doses 2 and 3, respectively, in control subjects) (**Figure 3C**). By Day 365, 100% of vaccinees still had detectable anti-SE36 IgG titres, with 39.4-fold change from Day 0 (*vs* 46%, 1.4-fold change in control arm).

#### 3.3.2 Protective epitopes on SERA5 one month after vaccination

The quality of the immune response was assessed by mapping the dominant epitopes in vaccinees’ sera against overlapping synthetic peptides covering the whole SE36 sequence (**Supplementary Figure 1C**). Box-whisker plots of normalised ODs for the 15 peptides in samples collected 4 weeks after Doses 2 and 3, show that reactivity was higher and broader in younger cohorts (*i.e*, 12–24-month-old > 5-10 years old > 21-45 years old) vaccinated with BK-SE36/CpG (**Supplementary Figures 1A, B**). Vaccinee sera predominantly reacted to peptides that lie in intrinsically unstructured regions (peptides 1-3, 7-9) of SE36 (**Supplementary Figure 1C**) (40). Control sera reacted poorly to all peptides spanning SE36.

#### 3.3.3 Incidence of clinical malaria one month post dose 2 in cohort 2

The trial was not designed to measure vaccine efficacy but an additional exploratory objective was to look at differences in malaria incidence. There were very few malaria episodes in Cohorts 1 (n = 4) and 3 (n = 1). Cohort 2, in which subjects received three vaccine doses prior to the rainy season, experienced more episodes of clinical malaria (defined as ≥ 5000 parasites/µL + fever, ≥ 38.0°C) with 5 episodes among 30 participants in the BK-SE36/CpG arm (5/30) compared with 8 episodes in 15 participants in the control arm (8/15) (rate ratio compared to control arm = 0.31, 95% CI 0.1-1.0, *p*-value = 0.055, likelihood ratio test). Analysis using the first episode produced similar results (BK-SE36/CpG: 4/30, 13% *vs* control: 6/15, 40%) (rate ratio compared to control arm = 0.26, 95% CI 0.07-0.92, *p* = 0.024, log-rank).

## 4 Discussion

In this study, the safety, reactogenicity and immunogenicity of three doses of the malaria vaccine candidate BK-SE36/CpG intramuscularly administered at Days 0, 28 and 112, were evaluated in 135 healthy African adults and children exposed to the parasite *Plasmodium falciparum* in Burkina Faso. This trial provides additional knowledge on the safety profile of BK-SE36/CpG that was previously tested in a two-dose regimen in a Phase 1a trial in Japanese malaria naïve adults (33). An age de-escalation design across three cohorts was chosen as this vaccine formulation has not been previously administered to malaria-exposed population. Moreover, even for the licensed CpG 1018-adjuvanted hepatitis B vaccine (41), or in clinical trials using CpG7909 (42–44), and CpG-ODN (K3) (45, 46), no safety data on the utility of TLR9-activating CpG-rich adjuvants was available in children.

The vaccine was well tolerated in all three cohorts. No subject experienced a SUSAR, Grade 3 vaccine-related AE, or AE that led to trial withdrawal. The five SAEs that occurred during the trial (52-226 days post vaccination) were all inpatient hospitalizations due to malaria.

AEs were more common in the vaccine arm, but were mostly Grade 1 events. The most common local symptoms were mild or moderate injection site pain or swelling; systemic symptoms were mostly headache and fever. All solicited vaccine-related AEs resolved spontaneously within 3 days after onset; unsolicited vaccine-related AEs resolved within 3 weeks. In all cohorts, the proportion of subjects that experienced AEFIs was higher in the BK-SE36/CpG arm than in the control arm. Most were of short duration, Grade 1 or Grade 2, except for two Grade 3 events (alanine aminotransferase increased and aspartate aminotransferase increased) in one subject in Cohort 1 (resolved 21 and 247 days from onset, respectively) unlikely to be related to vaccination. Four other Grade 3 AEs that occurred during the trial resolved 7-63 days from onset; all were unlikely to be or not related to vaccination.

Large variations were observed in haematology and biochemical tests and were considered to be not clinically significant. Due to theoretical concerns related to the potential of CpG ODN to evoke or unmask underlying autoimmune disease (31), 4 autoimmune markers were assessed. The use of ANA and anti-dsDNA as markers for autoimmune disease or inflammation has been reported in HEPLISAV trials (39). Although toxicity has not been observed in humans where CpG ODNs were used as vaccine adjuvants (47), these markers were assessed for monitoring the development of AEs: ANA, for autoantibodies against a nuclear component of the cell; anti-dsDNA antibodies, rare in healthy subjects but highly specific for the diagnosis of Systemic Lupus Erythematosus (SLE); ANCA, known marker for the diagnosis of vasculitis. As in all tests, the interpretation of their presence is to be taken in the light of clinical data as well as the sensitivity and specificity of the methods used for their detection. In ANCA, two fluorescence patterns have been reported and tested, cytoplasmic (c-ANCA, targeting a protein called proteinase 3) and perinuclear (p-ANCA, targets a protein called myeloperoxidase).

Frequent fluctuations or early elevations in ANA, anti-dsDNA and ANCA were observed in both the BK-SE36/CpG and control arm but none were considered to be associated with any clinically significant signs or symptoms. No evidence of autoimmune disease was noted in subjects that were positive for any of the markers. No subjects were also positive for at least two markers at the same time. The results are similar to the Phase 1a trial in malaria naïve adults where relatively non-specific and frequent fluctuations from baseline in ANA levels did occur in both the CpG and non-CpG groups (33). The suggestion (48) to use more specific markers, *e.g*. anti-dsDNA alone, and to check for other markers if anti-dsDNA is positive or if clinical symptoms are present might be considered, if logistically possible, in future, larger studies. It is also possible that infrequent AEs, not identified in this trial, may subsequently be detected with further investigation in larger Phase 2/3 trials or with long-term follow-up.

BK-SE36/CpG was immunogenic in all three cohorts. Despite the relatively small sample size, mean anti-SE36 antibody titres were higher in the BK-SE36/CpG arm compared to control arm. The highest response was observed in the youngest cohort. Anti-SE36 IgG antibodies were detected before vaccination in all arms across all cohorts suggesting seropositivity as a result of natural *P. falciparum* infection (49). This is also consistent with the age-dependency of naturally acquired IgG titres specific to SE36 (19). Notably, in the Solomon Islands and in Uganda, seropositivity in adults was 50%; and <10-20% in children (10-15 years old) (19, 20). Seropositivity in Burkina Faso appears to be higher: 48% for 12-24-month-old in Banfora (21); and 78%, 53%, and 27% for 21-45 years-old, 5-10 years-old and 12-24-month-old, respectively in Ouagadougou (this study). The high baseline antibody titres in Ugandan adults were presumed to limit the extent to which antibody responses can be boosted by BK-SE36 vaccination (20). This reduced responsiveness is thought to be due to immune tolerance.

Improved vaccine immunogenicity with the use of CpG-ODN (K3) was reflected in both the rapid seroconversion rates and the high titres obtained in all age cohorts. It is difficult to compare results since trials were conducted in different populations and dosing schedule. Nevertheless, if one compares 4 weeks post Dose 2 response in Ugandan adults (21-32 years-old) using BK-SE36 (20) and 21-45 years-old Burkinabe adults (Cohort 1) administered with BK-SE36/CpG, seroconversion was 22% *vs* 87%, respectively. In 12-24-month-old Burkinabe children residing in Banfora administered with BK-SE36, 79% and 94% seroconversion; and a 14- and 31-fold change from Day 0 was observed after Doses 2 and 3, respectively (21). In the same age group but residing in Ouagadougou and administered with BK-SE36/CpG, 100% seroconversion; and a 491- and 825-fold change from Day 0 were observed after Doses 2 and 3, respectively (this study). Thus, it appears that the use of CpG can overcome or break immune tolerance. Promising results were also reported for AMA1-C1/Alhydrogel ± CpG 7909 trial in Malian adults (43). Greater than 2-fold (2.5-3.2) increase in antibody response was seen in the group receiving CpG 7909 at all timepoints after second vaccination (*vs* non-CpG 7909 arm); albeit 11-14-fold higher antibody titres were observed in US naïve adults when CpG 7909 was added to AMA1-C1/Alhydrogel. Differences in the immunostimulatory functions of CpG ODNS was also observed (32).

In BK-SE36 vaccinees, a third dose resulted in higher immune responses, either 6- (21) or 4-months [this study] post Dose 1. Four weeks post Dose 3 resulted in highest anti-SE36 antibody titres in all cohorts. Moreover, in all cohorts, one year after the first vaccination the immune response for those who received BK-SE36/CpG was still high than at one-month post Dose 1.

There have been reports that increased immunogenicity did not always correlate with *in vitro* parasite growth inhibition (48, 50) or have an impact on parasite multiplication rates (51). For SE36, setting up a straightforward *in vitro* functional assay to demonstrate the neutralizing ability of these antibodies has been a challenge in terms of reproducibility, but epitope mapping studies has proven to be a valuable tool (40). The pooled affinity-purified antibodies from epitope mapping correlate well with ADCI. In all cohorts, sera from BK-SE36/CpG vaccinees predominantly reacted to synthetic peptides 7-9 spanning the SE36 protein. Sera from younger cohorts also showed broad reactivity to peptides 2-4, 13, and 15. Antibody-dependent cellular inhibition assay and challenge studies in squirrel monkeys have indicated that peptides 1-3 are protective epitopes; however, murine, squirrel monkey and other high titre Ugandan serum also do not rule out the presence of protective epitopes in other peptide regions of SE36 (40). Peptide 15 was also identified to bind vitronectin, a host molecule implicated for molecular camouflage (52). Peptides 1-3 and 7-9 corresponds to regions that are characterised as intrinsically unstructured (40). These results together with information on malaria incidence and fever (albeit limited in terms of small number of malaria cases observed) support further clinical evaluation of BK-SE36/CpG in a larger proof-of-concept study. At the time this trial was designed, the timing of vaccine schedules in relation to the transmission season as well as the SMC implementation were not fully appreciated (37, 53). Future trials will need to take these into consideration as additional factors that may also influence vaccine efficacy.

In conclusion, our results confirmed the safety profile of BK-SE36/CpG observed in malaria naïve adults living in Japan (33). The vaccine was immunogenic. This study is also the first to clinically evaluate CpG-ODN (K3) adjuvant in a paediatric population.

## Data availability statement

The original contributions presented in the study are included in the article/**Supplementary Material**. Further inquiries can be directed to the corresponding authors.

## Ethics statement

The studies involving humans were approved by National Ethical Committee (Comité d’Éthique pour la Recherche en Santé); National Regulatory Board, Burkina Faso; London School of Hygiene and Tropical Medicine Research Ethics Committee; Research Institute for Microbial Diseases Ethics Committee, Japan. The studies were conducted in accordance with the local legislation and institutional requirements. Written informed consent for participation in this study was provided by the participants’ legal guardians/next of kin.

## Author contributions

AO: Conceptualization, Investigation, Writing – original draft. EB: Conceptualization, Investigation, Writing – review & editing. NP: Conceptualization, Formal Analysis, Investigation, Methodology, Writing – original draft. SH: Conceptualization, Formal Analysis, Funding acquisition, Project administration, Writing – original draft. IN: Conceptualization, Investigation, Writing – review & editing. JS: Investigation, Writing – review & editing. GB: Investigation, Writing – review & editing. IS: Investigation, Writing – review & editing. AD: Investigation, Writing – review & editing. DH: Investigation, Writing – review & editing. AZO: Data curation, Methodology, Writing – review & editing. AK: Investigation, Writing – review & editing. SK: Project administration, Resources, Supervision, Writing – review & editing. AM: Methodology, Project administration, Writing – review & editing. SE: Methodology, Writing – review & editing. KI: Methodology, Writing – review & editing. TS: Conceptualization, Project administration, Resources, Writing – review & editing. FD’A: Conceptualization, Formal Analysis, Project administration, Writing – original draft. OL: Conceptualization, Funding acquisition, Resources, Supervision, Writing – review & editing. AT: Conceptualization, Investigation, Writing – review & editing. SC: Data curation, Formal Analysis, Methodology, Writing – original draft. TH: Conceptualization, Formal Analysis, Funding acquisition, Resources, Supervision, Writing – review & editing. SS: Funding acquisition, Investigation, Supervision, Writing – review & editing, Conceptualization, Formal Analysis.
