# Supplementary material for: Safety and immunogenicity of BK-SE36/CpG malaria vaccine in healthy Burkinabe adults and children: a phase 1b randomised, controlled, double-blinded, age de-escalation trial
Source: Front Immunol. 2023 Oct 16;14:1267372. doi: 10.3389/fimmu.2023.1267372 (PMC10613650; doi:10.3389/fimmu.2023.1267372)

Supplementary Material

Safety and immunogenicity of BK-SE36/CpG malaria vaccine in healthy Burkinabe adults and children: a phase 1b randomised, controlled, double-blinded, age de-escalation trial

Alphonse Ouédraogo, Edith Christiane Bougouma, Nirianne Marie Q. Palacpac, Sophie Houard, Issa Nebie, Jean Sawadogo, Gloria Damoaliga Berges, Issiaka Soulama, Amidou Diarra, Denise Hien, Amidou Z. Ouedraogo, Amadou T. Konaté, Seni Kouanda, Akira Myoui, Sachiko Ezoe, Ken J. Ishii, Takanobu Sato, Flavia D'Alessio, Odile Leroy, Alfred B. Tiono, Simon Cousens, Toshihiro Horii, Sodiomon B. Sirima

***Corresponding Author**:

Toshihiro Horii
horii@biken.osaka-u.ac.jp

Sodiomon B. Sirima
s.sirima@gras.bf

1 Supplementary Data

PROTOCOL SYNOPSIS

**Title**	Age de-escalation study to assess safety and immunogenicity of recombinant *E. coli* BK-SE36 malaria vaccine candidate formulated with CpG adjuvant administered intramuscularly in healthy malaria-exposed African adults and children living in Burkina Faso
**Trial Identifier**	NPC-SE36/001
**Principal Investigator**	Dr. Sodiomon Bienvenu Sirima, BA, MD, PhD<br>Institut de Recherche en Sciences de la Santé (IRSS)
**Active ingredient**	SE36 protein of *P. falciparum*
**Study population**	Healthy malaria exposed African children and adult subjects living in Burkina Faso:<br>21-45 years-old<br>5-10 years-old<br>12-24 months-old
**Trial Centre**	One centre in Ouagadogou, Burkina Faso
**Planned Trial Period**	Study Period (Months): 23 months<br>(Date of First Subject Enrolment: 23 May 2018<br>Date of Last Subject completed: 27 Apr 2020)
**Rationale**	**Background information on the disease to be treated**<br>Malaria is a parasitic disease with the highest disease burden on a global level. Despite progress in reducing the malaria burden, still an estimated 214 million cases occurred globally in 2015 with 438,000 deaths, the large majority in African children under 5 years old. A malaria vaccine is crucial in the face of continued high malaria transmission, increasing drug and insecticide resistance, and inadequate coverage of current control interventions. To date, only RTS,S, a pre-erythrocytic stage vaccine has shown a partial protection against malaria.<br><br>**Background information on the BK-SE36 vaccine**<br>There is a strong justification for blood-stage vaccines since protection by the anti-sporozoite RTS,S/AS01 vaccine candidate, currently the most advanced malaria vaccine, is not complete and long lasting. Asexual-stage parasites cause symptomatic malaria and blood-stage antigens are targets of acquired immunity. Controlling parasite density may reduce disease severity and *P. falciparum* gametocyte density, hence, potentially reducing disease transmission.<br><br>The *P. falciparum* serine repeat antigen-5 (SERA5) is an abundant blood-stage antigen secreted in large amounts into the lumen of the parasitophorous vacuole. It plays an essential role in the parasite life cycle and was among the first physiological substrate identified for a serine protease implicated for parasite egress.<br>A recombinant form of SERA5 N-terminal domain (SE36) was selected for clinical development on the basis of the following: (i)epidemiological studies showing high antibody titres that inversely correlate with malaria symptoms and severe disease; (ii) *in vitro* studies demonstrating induction of antibodies that are inhibitors of parasite growth, exert antibody-dependent complement-mediated lysis of schizonts, or antibody-dependent monocyte-mediated parasite growth inhibition; and (iii) animal studies demonstrating protection against *P. falciparum* challenge in non-human primates.

	SE36 was prepared under current Good Manufacturing Practice (cGMP) constraints and formulated with aluminium hydroxide gel (AHG) to yield BK-SE36. The safety and immunogenicity of BK-SE36 was demonstrated in a phase 1a trial in malaria naive Japanese adults; and in a phase 1b trial conducted in healthy subjects aged 6–32 years from a malaria endemic area in Northern Uganda. The trial promising results justified the conduct of a phase 1b trial of BK-SE36 in younger cohorts aged from 1 - 5 years old in Burkina Faso. The trial aimed to test the immune response in younger cohorts that has so far not been included in BK-SE36 malaria vaccine clinical trials. The trial results provided additional data on safety and immunogenicity.<br><br>**Trial rationale**<br>The immune response to BK-SE36 may still be improved with the use of DNA sequences containing CpG motifs that can selectively promote cellular and/or humoral immune responses. The phase 1b trial in Uganda demonstrated low sero-conversion in malaria exposed adults. The use of CpG K3 ODN may be one approach to broaden immune responses as a robust immune response may overcome immune tolerance or help immunocompromised individuals through the activation of multiple innate receptors that could target redundant pathways of innate responsiveness. A phase 1a clinical trial using BK-SE36/CpG was conducted in healthy adults in Japan where the vaccine was deemed safe and elicited antibody titres 3- to 4- fold higher as compared to BK-SE36 alone.<br><br>This study assessed BK-SE36/CpG safety and immunogenicity in a population exposed to malaria.
**Objectives**	**Primary**<br>Assess the safety and reactogenicity of 3 doses of malaria vaccine candidate BK-SE36 (100 µg SE36 + aluminium hydroxide gel) mixed with TLR9 ligand (CpG K3 ODN), via the intramuscular route, in healthy African adults and children exposed to the parasite *P. falciparum.* The adverse event grading for clinical abnormalities will be done according to the Brighton collaboration guidelines (www.brightoncollaboration.org). The primary objective is restricted to serious or severe adverse events.
	**Secondary**<br>• Assess the humoral immune response to the vaccine antigens administered intramuscularly by measuring the level of IgG in all subjects<br>• Assess the safety and reactogenicity of the BK-SE36/CpG vaccine restricted to mild or moderate adverse events.
	**Exploratory**<br>• Assess the quality of the humoral immune response on all participants by mapping the protective epitope(s) in SE36;<br>• To measure the preliminary vaccine efficacy against naturally occurring *P. falciparum* infection
**Trial design**	Double blind, single-dosage, randomised, controlled, age de-escalating, phase Ib clinical trial.

Number of Subjects (Planned and Analysed):

	BK-SE36 + CpG (100 µg SE36)			Control vaccine (Rabies)		
	**Cohort 1**	**Cohort 2**	**Cohort 3**	**Cohort 1**	**Cohort 2**	**Cohort 3**
Planned	30	30	30	15	15	15
Randomised	30	30	31	15	15	14
Received Dose 1	30	30	31	15	15	14
Received Dose 2	30	30	31	15	15	14
Received Dose 3	29	30	31	15	15	14
Total Withdrawn (Any reason)	1	0	0	0	0	1
Withdrawn due to lost to follow-up	1	0	0	0	0	1
Withdrawal of consent	0	0	0	0	0	0

Rationale for the number of doses: 2 vaccinations (28 days apart) + 1 booster dose. Immunogenicity results from a Phase 1a trial in Japan with BK-SE36/ CpG suggest that 2 administrations was enough for 100% seroconversion in malaria naïve population. Antibody titres were 3- to 4-fold higher than BK-SE36. However, a booster dose may be needed in malaria endemic areas and thus, inclusion of a booster dose at week 16 is intended to increase immunogenicity in the malaria exposed population.
Safety and immunogenicity of doses 21-days apart was confirmed in a phase 1a trial. A dosing interval of 28-days would be similar to schedules of inactivated vaccines.

Rationale for the age cohort: As no safety data exist in malaria endemic areas for the BK-SE36/CpG formulation, a cautious age de-escalation trial is planned keeping the age limit to 12 months. The Expanded Programme on Immunisation (EPI) vaccines are given up to 11 months, children below 12 months receiving EPI vaccines will be excluded to avoid any vaccine interference.

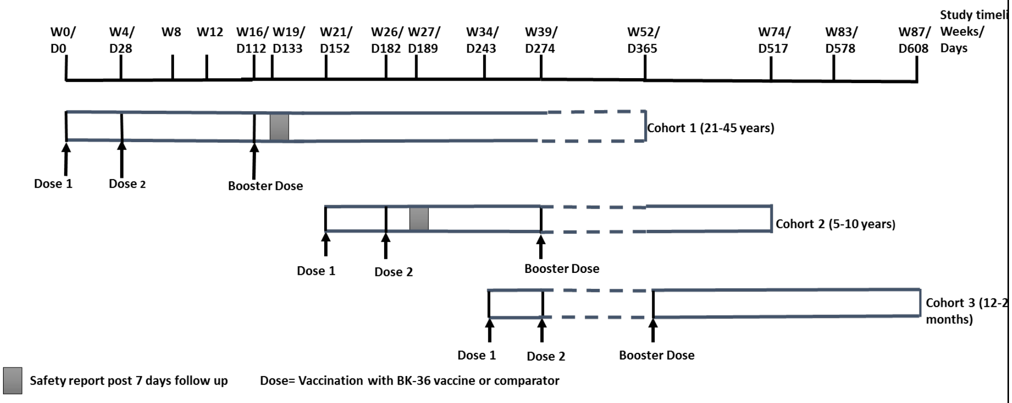

GO/No GO

The age de-escalation GO/No GO criteria will be based on safety criteria:
The No GO stopping rule will be: any SAE (Serious Adverse Event) possibly, probably or definitely related to vaccination or 50% or more of subjects having a Grade 3 adverse reaction persisting at Grade 3 for > 48 hours within 7 days following each vaccine dose (day of vaccination and 7 subsequent days). The threshold of 50% corresponds to a consensus decision in accordance with the African Malaria Network

	Trust (AMANET) Malaria vaccine guidelines and will be applied for the start of the vaccination of cohort 2 and 3.<br><br>For the age de-escalation to cohort 2, the Local Safety Monitor (LSM) and the Principal Investigator (PI) will review the safety report on data collected within 7 days after the third vaccination of cohort 1 and will authorise the age de-escalation if there is no safety concern. If there are any safety concerns, the unblinded safety report will be submitted to the members of the Independent Safety Monitoring Committee (ISMC) for their individual recommendations to the sponsor representative.<br><br>For the age de-escalation to cohort 3, a safety report will be prepared after the second vaccination of cohort 2 on the cumulative safety data from cohort 1 and cohort 2 collected within 7 days after the second vaccination of cohort 2. The blinded safety report will be directly submitted to the ISMC for their recommendation to the sponsor representative.<br><br>If any vaccinee experiences an SAE related to vaccination, then all the immunisations of the subjects in this group will be stopped. The vaccination will only be resumed upon the decision of the sponsor according to the recommendation of the LSM, PI and ISMC.<br>All safety data will be shared with the sponsor and sponsor's representative.<br><br>GO/No GO criteria for immunogenicity: There is no immunogenicity stopping rule with respect to moving ahead from cohort 1 to cohort 2 and from cohort 2 to cohort 3.
**Inclusion Criteria**	**Specific inclusion criteria for cohort 1 (adults 21-45 years old)**<br>• Female or male subjects aged 21 to 45 years inclusive at the time of the first vaccination<br>• Residing within the Ouagadougou health region and planning to stay for the study duration<br>• General good health based on medical history and clinical examination<br>• Written informed consent obtained before any trial procedure<br>• Female and male volunteers practicing/willing to practice contraceptive methods recommended by the national health system for at least four (4) weeks before the first vaccination (for female participants only) and up to four (4) weeks after the third vaccination.<br><br>**Specific inclusion criteria for cohort 2 (children 5-10 years old)**<br>• Female or male subjects aged 5 to 10 years inclusive at the time of first vaccination.<br>**Specific inclusion criteria for cohort 3 (children 12-24 months)**<br>• Female or male subjects aged 12 to 24 months inclusive at the time of first vaccination.<br>**Common inclusion criteria for cohorts 2 and 3**<br>• Residing within the Ouagadougou health region and planning to stay for the study duration<br>• Appear to be in generally good health based on malnutrition index and clinical and laboratory investigations<br>• Signed or thumb-printed informed consent obtained from the parent(s)/guardian(s) of the child. Where parent(s)/guardian(s) are not literate, the consent form will be countersigned by an impartial witness<br>• Subjects for whom the investigator believes that their parents/guardians can and will comply with the requirements of the protocol (e.g. return for follow-up visits) will be enrolled in the study. The trial period for each subject is 14 months (12 months + 8 weeks screening).

<table>
<tr><td></td><td></td></tr>
<tr><td>Non-inclusion criteria</td><td>Specific non-inclusion criteria for cohort 1
• Positive pregnancy test
• Currently breastfeeding
• Suspected or current known alcohol or drug abuse

Specific non-inclusion criteria for cohort 3
• Weight-for-age Z score of less than –3 or other relevant clinical signs of malnutrition

For cohort 1, 2 and 3
• Previous participation in any malaria vaccine trial
• History of blood transfusion within the last 3 months
• Symptoms, physical signs or laboratory values suggestive of systemic disorders, including renal, hepatic, cardiovascular, pulmonary, skin, immunodeficiency, psychiatric and other conditions, which could interfere with the interpretation of the trial results or compromise the health of the volunteers
• Any clinically significant laboratory abnormalities on screened blood samples outside the normal range, as defined at the clinical trial site. Specifically:
o Haemoglobin less than 8.0 g/dL,
o Serum creatinine concentration:
o Cohort 1 (21-45 years old):
▪ Female participants: serum creatinine concentration greater than 79.56 µmol/L
▪ Male participants: serum creatinine concentration greater than 88.40 µmol/L
o Cohort 2 (5-10 years old): serum creatinine concentration greater than 70.00 µmol/L
o Cohort 3 (12-24 month-old): serum creatinine concentration greater than 90.00 µmol/L
o Serum ALT concentration greater than 45 U/L,
o Low platelet count (< 100,000/mm^3)
• Immunosuppressive therapy (steroids, immune modulators or immune suppressors) within 3 months prior to recruitment. (For corticosteroids, this will mean prednisone, or equivalent, ≥ 0.5 mg/kg/day. Inhaled and topical steroids are allowed.)
• Any confirmed or suspected immunosuppressive or immunodeficiency condition based on medical history and physical examination (No testing will be done for HIV)
• A family history of congenital or hereditary immunodeficiency
• History of auto immune disease
• Major congenital defects
• Subjects with splenectomy
• History of anaphylaxis or known severe hypersensitivity to any of the vaccine components (adjuvant or antigen or excipient)
• Administration of gamma globulin: 4 weeks prior to and after each vaccination; if administration is necessary during the study period, the volunteer will be withdrawn from the study
• Planned administration/administration of a vaccine not foreseen by the study protocol within 30 days of the first dose of vaccine(s)</td></tr>
</table>

<table>
<tr><td></td><td>

Use of any investigational or non-registered drug or vaccine within 30 days preceding the first dose of study vaccine, or planned use during the study period
Current participation in another clinical trial, or within 12 weeks of this study
Any other finding which in the opinion of the investigators would increase the risk of an adverse outcome from participation in the trial or result in incomplete or poor quality data.

</td></tr>
<tr><td>Holding and stopping rules</td><td>
The trial may be placed on safety hold for the following reasons:

On advice of the ISMC
On advice of the LSM.
On advice of the PI.
One or more participants experience a serious adverse event (SAE) that is determined to be possibly related to BK-SE36/CpG vaccine administration.
In case of an SAE possibly related to BK-SE36/CpG vaccination, the immunisation of the remaining subjects will be immediately (but not finally) discontinued until the decision of the sponsor or the sponsor’s representative according to the LSM, PI and ISMC recommendations. The LSM and PI will arrange a meeting within 48h following the SAE to assess whether the event was unrelated or related to the vaccine. They will recommend stopping, or pausing or continuing the immunisation to the ISMC. The ISMC members can individually inform the sponsor’s representative by email or at its discretion call for a TC to discuss any safety concerns within 48h of receipt of the report from LSM and PI. The final decision on stopping, or pausing or continuing the vaccination will belong to the sponsor taking into account recommendations of the LSM, PI and ISMC.

The trial will be stopped if the following occurs:

One or more participants experience a Suspected Unexpected Serious Adverse Reaction (SUSAR) that is related to the study vaccine’s administration.

</td></tr>
<tr><td rowspan="5">Investigational products</td><td>BK-SE36 is recombinant SE36 protein expressed in E. coli, adsorbed to aluminium hydroxide gel (AHG) and further adjuvanted with CpG.</td></tr>
<tr><td>Form
Lyophilised; reconstitution with CpG solution prior to administration</td></tr>
<tr><td>Dosage
100 µg/mL P. falciparum SE36 protein</td></tr>
<tr><td>Adjuvant
Aluminium: 1000 µg/mL
CpG-ODN (K3) solution: 1000 µg /mL
Ratio of protein to adjuvant= 1:10:10</td></tr>
<tr><td>Route of administration
Intramuscular</td></tr>
<tr><td rowspan="2">Control product</td><td>Rabies vaccine</td></tr>
<tr><td>Route of administration
Intramuscular</td></tr>
<tr><td>Vaccination Schedule</td><td>
<table>
<tr><td></td><td colspan="6">Study cohorts</td></tr>
<tr><td></td><td colspan="2">1. Adults aged 21-45 years old</td><td colspan="2">2. Children aged 5-10 years old</td><td colspan="2">3. Children aged 12-24 months old</td></tr>
<tr><td></td><td>Control arm</td><td>Vaccine arm</td><td>Control arm</td><td>Vaccine arm</td><td>Control arm</td><td>Vaccine arm</td></tr>
<tr><td>Schedule</td><td>n=15</td><td>n=30</td><td>n=15</td><td>n=30</td><td>n=15</td><td>n=30</td></tr>
<tr><td>Day 0</td><td>Rabies</td><td>BK-SE36/CpG</td><td>Rabies</td><td>BK-SE36/CpG</td><td>Rabies</td><td>BK-SE36/CpG</td></tr>
<tr><td>Week 4</td><td>Rabies</td><td>BK-SE36/CpG</td><td>Rabies</td><td>BK-SE36/CpG</td><td>Rabies</td><td>BK-SE36/CpG</td></tr>
<tr><td>Week 16</td><td>Rabies</td><td>BK-SE36/CpG</td><td>Rabies</td><td>BK-SE36/CpG</td><td>Rabies</td><td>BK-SE36/CpG</td></tr>
</table>
</td></tr>
</table>

**Number of scheduled Visits**	11 clinic visits on site (+ 18 contact visits).
**Follow-up duration**	Total duration: 12 months following the first vaccination.
**Serology Schedule**	At Week-4 (screening), Day 0 (before first administration), Week 1, 4 (before second administration), 5, 8, 16 (before booster dose), 17, 20, 26, and 52.
**Sample size justification**	The sample size is calculated to address the primary objective (safety). In each cohort (N=29), if the underlying risk of an SAE is 10%, then the probability of seeing at least one SAE is 95%. If the risk is 5% then the probability of seeing at least one SAE is 77%. With 87 vaccinees the probability of detecting at least one SAE is >99% if the risk is 10%, is 99% if the risk is 5% and 58% if the risk is 1%.
**Endpoints**	**Primary**<br>• Occurrence of solicited adverse events considered related to vaccination (possibly, likely or definitely) and that are severe (Grade 3) within 7 days following vaccination (day of vaccination and 7 subsequent days)<br>• Occurrence of unsolicited adverse events (AEs) considered related to vaccination (possibly, likely or definitely) and that are severe (Grade 3) within 28 days following each vaccination (day of vaccination and 28 subsequent days), according to the Medical Dictionary for Regulatory Activities (MedDRA classification)<br>• Occurrence of serious adverse events (SAEs) at any point during the study period, according to the Medical Dictionary for Regulatory Activities (MedDRA classification)
	**Secondary**<br>• Anti-SE36 protein IgG antibody titre at Day 0, Week 4, 8, 16, 20 and 52.<br>• Occurrence of solicited adverse events reactions considered related to vaccination (possibly, likely or definitely) and that are mild or moderate (Grade 1 or Grade 2) within 7 days following vaccination (day of vaccination and 7 subsequent days)<br>• Occurrence of unsolicited adverse events (AEs) considered related to vaccination (possibly, likely or definitely) and that are mild or moderate (Grade 1 or Grade 2) within 28 days following each vaccination (day of vaccination and 28 subsequent days), according to the Medical Dictionary for Regulatory Activities (MedDRA classification)
	**Exploratory**<br>• Determining protective epitopes on SE36 one month after booster dose (Week 20)<br>• Incidence of clinical malaria from Day 56 to Day 365
**Primary evaluation criteria**	The safety profile will be assessed by the following criteria:<br>• Immediate reactogenicity (reactions within 60 minutes after each vaccination)<br>• Local and systemic reactogenicity measured from Day 0 to Day 7 after each vaccination<br>• Any unsolicited adverse event within one month after each vaccination<br>• Any Serious Adverse Event (SAE) occurring throughout the study duration starting from the first immunisation.<br>The relationship of the adverse event to the vaccine will be established by the investigator as definitely, probably, possibly, unlikely related, or not related.<br>• Occurrence of clinically significant hematological and/or biochemical abnormalities by laboratory testing, one week and four weeks after each

	vaccination, Week 26 and 52 in reference with the baseline before the first dose, by measuring:<br>o RBC, hemoglobin, MCV, MCH, MCHC, platelets, ESR and WBC with differential counts<br>o AST, ALT, total bilirubin, creatinine<br>• Occurrence of clinically significant change in assessments of anti-dsDNA, anti-neutrophil cytoplasmic antibody (p- and c-ANCA), anti-nuclear antibody (ANA) at Day 0, Weeks 20 and 52.
**Secondary evaluation criteria**	• The humoral immune response to vaccination will be assessed by measuring the titre of SE36 specific IgG by ELISA on samples obtained before first vaccination at Day 0 and at Weeks 4, 8, 16, 20 and 52<br>• The safety profile will be assessed as described in the primary evaluation criteria
**Exploratory evaluation criteria**	• Mapping of protective epitope(s) by ELISA against overlapping peptides derived from the SE36 protein at Week 20<br>• Incidence of clinical malaria assessed by microscopic examination of thick and thin blood smears in the event of fever (temperature ≥ 38.0°C) or history of fever in the past 24 hours, starting from Day 56 to Day 365
**Statistical methods**	Interim Analysis<br>In order to obtain a preliminary assessment of the safety and immunogenicity of the vaccine without waiting until the end of the trial, an analysis of data up to and including visit 27 (28 days after the booster dose) will be performed for each cohort, after cohort 3 has completed visit 27. A statistician independent of the trial will perform the analysis to maintain the blinding of trial personnel. The statistician will generate simple, summary descriptive statistics by trial arm as follows:<br><br>- numbers of adverse events possibly, probably or definitely related to vaccination,<br>- the proportions of participants experiencing such events<br>- the proportions of participants with detectable anti BK-SE36 IgG titres<br>- geometric mean titres among participants with detectable titres<br><br>No formal statistical testing will be performed to compare the trial arms<br><br>Final analysis<br>The safety analysis population will include all participants who received at least one injection. Data from individuals who do not complete the full follow-up period will be included up until drop out/withdrawal.<br>Immunogenicity analyses will include all subjects who received at least one injection and will be performed on all available data at each-time point.<br>Statistical analyses will be exploratory, as the design is not powered to demonstrate statistically significant differences in outcomes.<br>Categorical variables will be summarised by trial arm as percentages and 95% confidence intervals. Geometric means and 95% confidence intervals of the antibody titres will be determined by trial arm. Continuous variables other than titres and concentrations will be summarised by trial arm and means, SEM, Medians, Minimums, Maximums, and inter-quartile ranges reported. The proportion of subjects that received the vaccine without experiencing a Grade 3 adverse event or SAE will be reported with exact binomial 95% confidence intervals. Malaria incidence by trial arm will be calculated as the number of malaria episodes divided by the person time of follow-up.

Schedule of Study Events

**Visit**	**1**	**2**	**3 - 8 Contact Visits**	**9**	**10**	**11-16 Contact Visits**	**17**	**18**	**19**	**20-25 Contact Visits**	**26**	**27**	**28**	**29**	**Uv***
Timelines Days	D-28-0	D0	D1-D6	D7	D28	D29-34	D35	D56	D112	D113-118	D119	D140	D182	D365	
Time window Days		±1D			±2D		±2D	±2D	±7D		±2D	±2D	±2D	±2D	
Timelines Weeks	W-4	W0		W1	W4		W5	W8	W16		W17	W20	W26	W52	
Description	Screening	Vac 1	Vac 1 +1-6	Vac 1 +7	Vac 2	Vac 2 +1-6	Vac 2 +7		Vac 3	Vac 3 +1-6	Vac 3 +7				
Eligibility Criteria	X														
Informed consent	X														
Inclusion/non-inclusion Criteria	X														
Medical history	X														
Physical examination	X	X		X	X		X	X	X		X	X	X	X	X
Contraindication Review		X			X				X						
Prior and concomitant therapy	X	X			X				X						
Randomisation		X													
Vaccination/ Immediate surveillance		X			X				X						
Non-serious Adverse Event (AE)		X	X	X	X	X	X	X	X	X	X	X	X	X	X
Serious Adverse Event (SAE)	To be reported at any time during the trial														
Blood sampling	X	X		X	X		X	X	X		X	X	X	X	X
Pregnancy serum test	X	X			X				X						
Laboratory safety	X	X		X	X		X	X	X		X	X	X	X	X[a]
SE36 IgG		X			X			X	X			X		X	X[b]
dsDNA, ANCA, ANA antibody		X										X		X	
Epitope mapping								(X)				X			
Blood smear for malaria infection		X			X			X	X			X		X	X
Blood smear for parasite polymorphism analysis†		X			X			X	X			X		X	X
T cell cytokines †		X						X				(X)		(X)	
miRNA †		X						X				(X)		(X)	
GIA †								(X)			X	(X)			
Total blood sampling per visit in mL	6	10		6	10		6	10	10		6	10	6	10	10

†=Blood was collected, however results were not encoded in the database,*UV= unscheduled visits, **a** =Blood was collected when deemed necessary by the investigator.
b=In case of clinical malaria occurring after one month post second vaccination, one blood sampling was done at the time of diagnosis and another one, one week after (at resolution of) the malaria event, **( )**= only if blood sample was available, least in order of priority. Maximum amount of blood 10 mL per subject per visit

2 Supplementary Table and Figures

Supplementary Table 1. Summary table of AEs occurring on days 0 to 28 post-vaccination

	**All AEs**		**AEs related to vaccination***		**AEs not related to vaccination**	
	**BK-SE36/ CpG n=91**	**Control (rabies) n=44**	**BK-SE36/ CpG n=91**	**Control (rabies) n=44**	**BK-SE36/ CpG n=91**	**Control (rabies) n=44**
**All AEs**						
Number of events	334	127	119	22	215	105
Number (%) of subjects	83 (91%)	37 (84%)	35 (38%)	6 (14%)	48 (53%)	31 (70%)
**SAEs**						
Number of events	0	0	0	0	0	0
Number (%) of subjects	0 (0%)	0 (0%)	0 (0%)	0 (0%)	0 (0%)	0 (0%)
**Grade 3 events**						
Number of events	5	0	0	0	5	0
Number (%) of subjects	3 (3%)	0 (0%)	0 (0%)	0 (0%)	3 (3%)	0 (0%)
**Grade 1 and 2 events**						
Number of events	329	127	119	22	210	105
Number (%) of subjects	83 (91%)	37 (84%)	35 (38%)	6 (14%)	48 (53%)	31 (70%)

*possibly probably or definitely

Supplementary Table 2. Proportion of participants who received 3 vaccine doses without experiencing a Grade 3 vaccine-related adverse event.

	Cohort 1		Cohort 2		Cohort 3	
	**BK-SE36/CpG**	**Control (rabies)**	**BK-SE36/CpG**	**Control (rabies)**	**BK-SE36/CpG**	**Control (rabies)**
Number of participants receiving all three vaccine doses	29	15	30	15	31	14
Number of Grade 3 adverse events possibly, probably, or definitely related to vaccination	0	0	0	0	0	0
Number (%) of participants experiencing a Grade 3 adverse event possibly, probably, or definitely related to vaccination	0 (0%)	0 (0%)	0 (0%)	0 (0%)	0 (0%)	0 (0%)
Number (%) of participants not experiencing a Grade 3 adverse event possibly, probably, or definitely related to vaccination	29 (100%)	15 (100%)	30 (100%)	15 (100%)	31 (100%)	14 (100%)
(95% CI)	(88 to 100%)	(78 to 100%)	(88 to 100%)	(78 to 100%)	(89 to 100%)	(77 to 100%)

Supplementary Table 3. Line listing of Grade 3 events throughout the trial.

Participant ID	Vaccine arm	MedDRA PT term	Start date	End date	Vaccine doses received	Days since last vaccine dose	Grade	Outcome	Relatedness to vaccine
Adverse events following immunization (AEFIs)									
SEK-A08	BK-SE36/CpG	Alanine aminotransferase increased	04 Oct 2018	25 Oct 2018	3	7	3	Resolved	Unlikely
SEK-A08	BK-SE36/CpG	Aspartate aminotransferase increased	04 Oct 2018	08 Jun 2019	3	7	3	Resolved	Unlikely
Other AEs occurring at any time during the trial									
SEK-C13	BK-SE36/CpG	Haemoglobin decreased	27 Apr 2019	04 May 2019	2	84	3	Resolved	Unlikely
SEK-T44	BK-SE36/CpG	Haemoglobin decreased	13 Jul 2019	14 Sep 2019	2	28	3	Resolving	Unlikely
SEK-T33	BK-SE36/CpG	Alanine aminotransferase increased	28 Sep 2019	19 Oct 2019	3	28	3	Resolved	Not related
SEK-T33	BK-SE36/CpG	Aspartate aminotransferase increased	28 Sep 2019	19 Oct 2019	3	28	3	Resolved	Not related

Supplementary Table 4. Line Listing of unsolicited events related to vaccination and occurring within 28 days of vaccine dose.

Participant ID	Vaccine arm	MedDRA PT term	Grade	Duration (days)	Relatedness to vaccine
Cohort 1, Dose 1					
SEK-A05	Control	Asthenia	1	1	Possibly
SEK-A13	Control	Injection site pain	1	<1	Definitely
SEK-A19	BK-SE36/CpG	Asthenia	1	2	Probably
SEK-A46	BK-SE36/CpG	Blood creatinine increased	1	21	Possibly
SEK-A16	BK-SE36/CpG	Injection site pain	1	2	Definitely
SEK-A19	BK-SE36/CpG	Pain	1	2	Probably
Cohort 1, Dose 2					
SEK-A05	Control	Back pain	1	10	Possibly
SEK-A37	BK-SE36/CpG	Dizziness	1	<1	Possibly
SEK-A14	BK-SE36/CpG	Headache	1	1	Possibly
Cohort 1, Dose 3					
SEK-A05	Control	Back pain	1	1	Possibly
Cohort 3, Dose 1					
SEK-T38	Control	Diarrhoea	1	1	Possibly
SEK-T24	Control	Injection site pain	1	<1	Definitely
SEK-T06	BK-SE36/CpG	Pyrexia	1	<1	Possibly
SEK-T21	BK-SE36/CpG	Pyrexia	1	<1	Probably
SEK-T23	BK-SE36/CpG	Pyrexia	1	1	Probably
SEK-T37	BK-SE36/CpG	Pyrexia	1	1	Probably
Cohort 3, Dose 3					
SEK-T15	Control	Diarrhoea	1	1	Possibly
SEK-T06	BK-SE36/CpG	Diarrhoea	1	2	Possibly
SEK-T22	BK-SE36/CpG	Pyrexia	1	1	Probably

frontiers

3 Supplementary Figures

Supplementary Figure 1. Box-whisker plots of normalised ODs for the 15 peptides covering SE36. Protective epitopes on SE36 one-month post Dose 2 and 3.

(A) 1-month post Dose 2

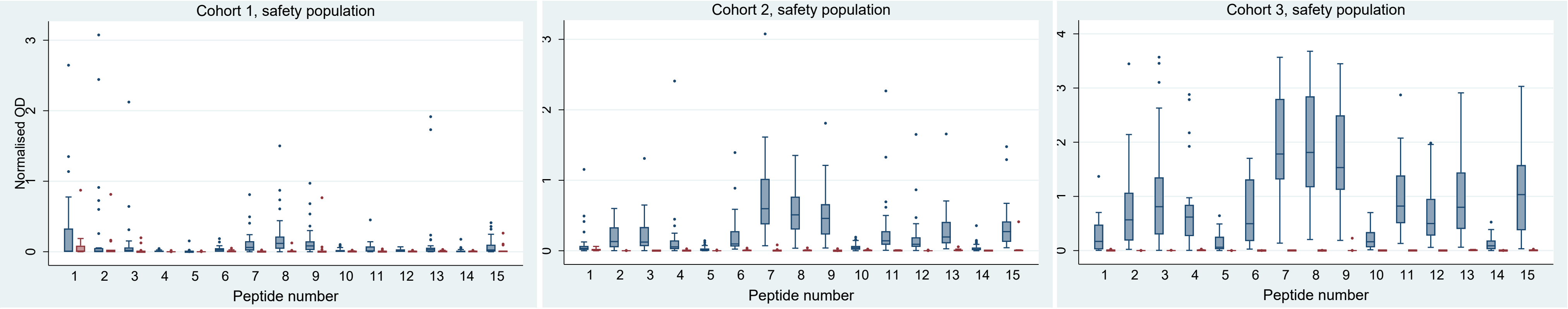

(B) 1-month post Dose 3

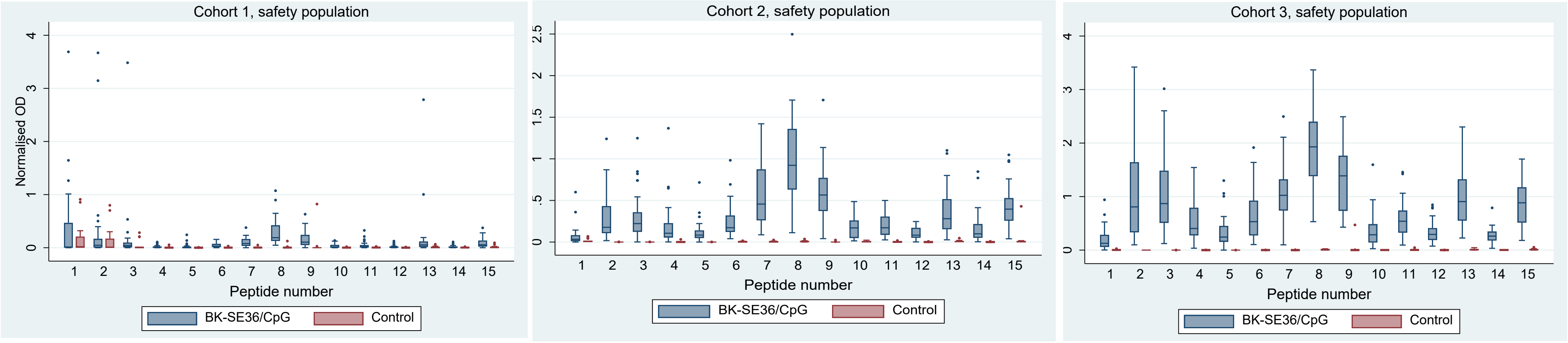

(C) Schematic representation of overlapping synthetic peptides covering the whole sequence of SE36

(*Yagi M, et al. PLoS One. 2014;9(6):e98460. doi:10.1371/journal.pone.0098460*))

Synthetic peptides range from 40-42 amino acids. In a previous protein structure-activity study [Yagi et al., 2014], peptides for the octamer and serine rich region did not show any ability to form rigid, typical secondary structures. Although the repeat number of octamer motifs varies depending on different *P. falciparum* strains the basic motif sequences are well conserved. A conserved parasite inhibitory epitope has been mapped on amino acids 17-73 using a murine monoclonal antibody. Using Ugandan high titre sera, affinity purified antibodies specifically recognising sequences from peptides 1-3, showed high antibody-dependent cellular inhibition activity. However, these regions may not be the sole protective epitopes since SE36 vaccinated mouse serum pool reacted broadly with a number of regions/peptides and correspondingly have high parasite inhibitory activity at comparable antibody concentrations. Position 178 corresponds to the polyserine sequence region.

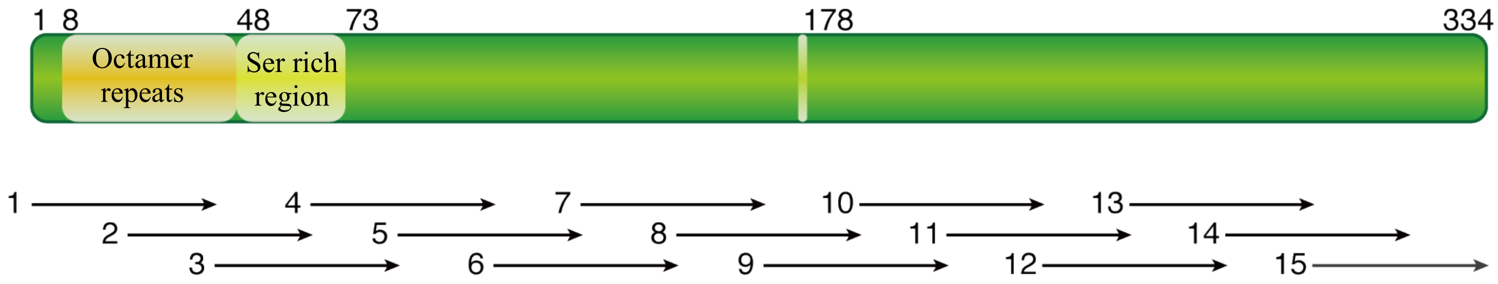

Supplement: Supplementary file 1 [file DataSheet_1.pdf]
